# Computational Modeling for Neuropsychological Assessment of Bradyphrenia in Parkinson’s Disease

**DOI:** 10.3390/jcm9041158

**Published:** 2020-04-18

**Authors:** Alexander Steinke, Florian Lange, Caroline Seer, Merle K. Hendel, Bruno Kopp

**Affiliations:** 1 Department of Neurology, Hannover Medical School, Carl-Neuberg-Straße 1, 30625 Hannover, Germany; 2 Behavioral Engineering Research Group, KU Leuven, Naamsestraat 69, 3000 Leuven, Belgium; 3 Movement Control & Neuroplasticity Research Group, Department of Movement Sciences, KU Leuven, Tervuursevest 101, 3001 Leuven, Belgium; 4 LBI - KU Leuven Brain Institute, KU Leuven, 3000 Leuven, Belgium

**Keywords:** computational modeling, reinforcement learning, Parkinson’s disease, dopamine, bradyphrenia, Wisconsin Card Sorting Test

## Abstract

The neural mechanisms of cognitive dysfunctions in neurological diseases remain poorly understood. Here, we conjecture that this unsatisfying state-of-the-art is in part due to the non-specificity of the typical behavioral indicators for cognitive dysfunctions. Our study addresses the topic by advancing the assessment of cognitive dysfunctions through computational modeling. We investigate bradyphrenia in Parkinson’s disease (PD) as an exemplary case of cognitive dysfunctions in neurological diseases. Our computational model conceptualizes trial-by-trial behavioral data as resulting from parallel cognitive and sensorimotor reinforcement learning. We assessed PD patients ‘on’ and ‘off’ their dopaminergic medication and matched healthy control (HC) participants on a computerized version of the Wisconsin Card Sorting Test. PD patients showed increased retention of learned cognitive information and decreased retention of learned sensorimotor information from previous trials in comparison to HC participants. Systemic dopamine replacement therapy did not remedy these cognitive dysfunctions in PD patients but incurred non-desirable side effects such as decreasing cognitive learning from positive feedback. Our results reveal novel insights into facets of bradyphrenia that are indiscernible by observable behavioral indicators of cognitive dysfunctions. We discuss how computational modeling may contribute to the advancement of future research on brain–behavior relationships and neuropsychological assessment.

## 1. Introduction

Neuropsychological impairments are well-documented in idiopathic Parkinson’s disease (PD; reviewed in [1]). The main pathological characteristic of PD is the decline of dopaminergic cells in the substantia nigra pars compacta (SNpc) [2,3]. Braak’s theory [2,4] characterizes disease progression, which is divided into six different stages. Early stages of PD are characterized by non-motor symptoms, including a number of neuropsychological impairments (reviewed in [1,5,6]). At later stages, the death of dopaminergic cells in the SNpc correlates with the emergence of motor symptoms, the presence of which typically lead to the diagnosis of the disease. According to Braak’s theory, dementia arises as the last Braak stages are reached. PD is treated by dopamine (DA) replacement therapy, which is adjusted to alleviate motor symptoms to the best possible motility. Thus, systemic DA replacement aims at substituting the missing DA in the dorsal striatum (in which the dopaminergic cells from the SNpc have their axonal terminals). However, titrating systemic DA replacement solely at the best possible motility may incur potential side effects. That is, optimal DA replacement in the nigro-striatal DA system may be associated with DA overdosing in meso-limbic and/or meso-cortical DA systems, thereby potentially inducing medication-related neuropsychological impairments [1,7,8,9,10,11,12].

A cardinal motor symptom of many PD patients is bradykinesia, which might be best summarized as ‘slowness of movement’ [13,14]. The neuropsychological equivalent of bradykinesia in PD is bradyphrenia, i.e., ‘slowness of thought’ [15,16,17,18,19,20,21,22]. However, bradyphrenia is not synonymous with slowed behavioral indicators of processing speed (e.g., response times in laboratory tasks), which would necessarily originate from both bradyphrenia and bradykinesia. Bradyphrenia has previously been described as cognitive akinesia [23], and we refer to it here as ‘inflexibility of thought’. As such, PD-related bradyphrenia is typically studied by a number of neuropsychological tests that are targeting covert aspects of attentional flexibility [24,25,26]. Of major interest for the current study are the behavioral abnormalities on the Wisconsin Card Sorting Test (WCST) [5,6,27], which are typically considered as behavioral evidence indicating attentional inflexibility in patients with PD [28]. Although these observable WCST indicators may provide well-documented sensitive markers of PD-related bradyphrenia, they yet lack specificity because similarly impaired WCST indicators occur in other neurological diseases [29,30,31,32,33,34] and also in psychiatric disorders [35,36,37,38].

**Figure 1:**
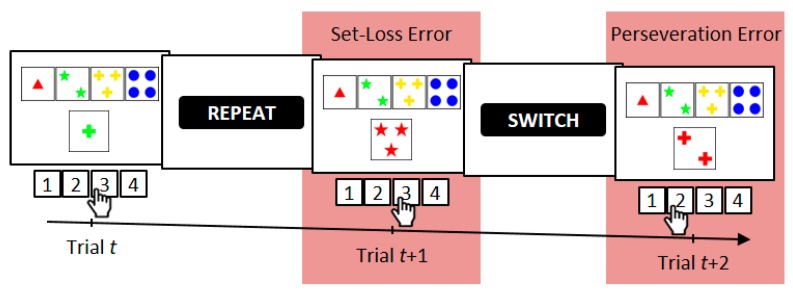
An exemplary trial sequence on the computerized Wisconsin Card Sorting Test (WCST) [27,45,46,47,48]. On Trial *t*, a stimulus card (one green cross) could be sorted by the color category (inner left key card, response 2), the number category (far left key card, response 1), or the shape category (inner right key card, response 3). In the current example, the shape category was applied as indicated by observing response 3. A positive feedback (i.e., the visually presented word “REPEAT”) indicated that this sort was correct, implying that the shape category should be repeated on the upcoming trials. However, on Trial *t*+1, the number category was applied, as indicated by observing response 3. Erroneous switches of the applied category after positive feedbacks are referred to as a set-loss errors. A subsequently presented negative feedback stimulus (i.e., the visually presented word “SWITCH”) announced that that sort was incorrect, implying that a category switch is required. On Trial *t*+2 of the current example, the number category was repeated, as indicated by observing response 2. Erroneous repetitions of categories following negative feedbacks are referred to as perseveration errors.

On the WCST [27,39,40,41], participants are required to sort stimulus cards to key cards according to a periodically changing category, which is one of three rival categories (see Figure 1). Behavioral WCST indicators are typically the number of perseveration errors (i.e., erroneous category repetitions following negative feedback) and the number of set-loss errors (i.e., erroneous category switches following positive feedback) [27]. Performance on the WCST can be conceptualized as feedback-driven learning [42,43,44] because in order to identify the prevailing category, participants have to rely on feedback about the correctness of their current sorts: Positive feedback indicates that the executed sort was correct, whereas negative feedback indicates that the executed sort was incorrect. We showed in an earlier study [43] that WCST feedback affects both the cognitive selection of sorting categories and the sensorimotor selection of a particular response. We thus conceptualized feedback-driven learning on the WCST as occurring at two distinguishable levels in parallel, i.e., as sensorimotor learning at the lower level (i.e., which response to execute) and as cognitive learning at the higher level (i.e., which category to apply).

Putatively related to their lack of specificity, the underlying neural mechanisms of these behavioral WCST indicators remain poorly understood [30,34]. Here, we suggest that progress with regard to WCST-derived brain–behavior relationships does not primarily depend on further improvements at the neuroanatomical (or neurofunctional) level. In our opinion, such progress primarily depends on further improvements at the hitherto much neglected behavioral level. These improvements include (though are not limited to) applying more elaborate data analysis methods such as computational modeling techniques [42,48,49,50,51,52].

The computational approach allows specifying learning processes that are assumed to underlie observable behavior. These learning processes can be conceptualized mechanistically, and they act together in explicitly defined ways. Computational models allow decomposing the finally expressed overt behavior on neuropsychological tests such as the WCST into interacting covert components. Hence, computational modeling of WCST performance allows quantifying some of the contributing learning mechanisms. The pursuance of the computational approach promises to allow investigating associations between explicitly defined learning processes and neural mechanisms [49,53].

Here, we applied the parallel reinforcement-learning (RL) [54,55,56,57,58,59,60] model of trial-by-trial behavior on the WCST, which we introduced in a recent publication [44]. Since learning on the WCST can be conceptualized as feedback driven, RL represents a natural approach for modeling dynamic changes in behavior on the WCST. Our RL model comprises distinct learning from positive feedback and from negative feedback. The reason for the distinction between positive and negative learning is that DA-midbrain signaling may code positive and negative learning in different ways due to the potential reward-quality of positive feedback [61,62,63]. We also incorporated a simple retention mechanism [64,65], which ensured that what was learned from feedback stimuli on a particular WCST trial remained available in mind for short periods of time. Furthermore, we conceptualized WCST performance at two parallel, yet distinct levels of learning [43,44]: The higher-level cognitive learning (which may also be referred to as cortical, declarative, or goal-directed) is completed by lower-level sensorimotor learning (which may also be referred to as striatal, procedural, or automatic). In the context of the WCST, cognitive learning considers objects of thought (i.e., which category to apply on a particular trial) that guide the selection of task-appropriate responses. Sensorimotor learning bypasses these objects of thought as it is solely concerned with selecting responses (see Figure 1): Responses that were followed by positive feedback tend to be repeated, whereas responses that were followed by negative feedback tend to be avoided on upcoming trials.

Our study was directed toward two main goals. The first goal was advancing neuropsychological assessment of bradyphrenia/attentional inflexibility in PD by expanding purely observable indicators, such as perseveration and set-loss errors on the WCST, through a computational approach. The second goal was characterizing the specific learning dysfunctions that are associated with disease pathology and/or DA replacement therapy in patients with PD. In order to achieve these goals, we studied patients with PD and matched healthy control (HC) participants twice on a computerized version of the WCST (cWCST) [27,45,46,47,48]. Patients with PD were assessed ‘on’ DA medication and ‘off’ DA medication (i.e., after withdrawal of DA medication).

## 2. Materials and Methods

### 2.1. Procedure

The relationship between PD pathology and cWCST performance was studied in a between-subjects design, comparing PD patients and HC participants. The effect of DA replacement therapy on cWCST performance was studied in a within-subjects design. PD patients were assessed during two testing sessions; once with their usual DA replacement therapy (‘on’ medication) and once after withdrawal of DA replacement therapy (‘off’ medication). To account for a potential effect of the repeated assessment on cWCST performance, we also assessed HC participants at two testing sessions, and we included the testing session as an additional within-subjects factor in the analyses. Note that analyses of first testing sessions were reported by [48,66].

Performance on the cWCST was first analyzed by means of conditional error probabilities. Second, we analyzed cWCST performance by means of computational modeling, i.e., we implemented the parallel reinforcement-learning model [44].

### 2.2. Participants

The initial sample of PD patients comprised 44 inpatients and outpatients with idiopathic PD referred to the department of Neurology at Hannover Medical School. Patients were diagnosed by experienced neurologists in the field of movement disorders. Patients with any severe neurological or psychiatric condition other than PD, or a history of neurosurgical therapy were not considered for this study. One patient was excluded due to antidepressant medication. We excluded patients who were assessed only once (*n* = 22) and patients who completed less than half of the cWCST (i.e., less than 20 switches of the correct category) in at least one of the testing sessions (*n* = 5), resulting in a final sample of *N* = 16 (11 female) PD patients. Table A1 displays PD patients’ demographic, clinical, and psychological data. Table A2 provides information about PD patients’ DA replacement therapy. None of the PD patients received infusional therapy at the time of testing. PD patients’ median Hoehn and Yahr stage was 2 (range: 1–3). Ten patients were tested first ‘off’ medication and six patients were tested first ‘on’ medication. The median time between the two testing sessions of PD patients was 2 days (range: 1–29). The median time of withdrawal from usual DA replacement therapy at the start of the testing session ‘off’ medication was 14 h (range: 4–177). The severity of clinical motor symptoms ‘off’ and ‘on’ medication was assessed by means of the Unified Parkinson’s Disease Rating Scale-part III (UPDRS III). PD patients’ mean UPDRS III scores were higher ‘off’ than ‘on’ medication, indicating an increase of motor symptoms after the withdrawal from DA replacement therapy (see Table A1).

Thirty-six participants without neurological or psychiatric diseases served as an HC group. One of these participants had to be excluded due to inability of performing the cWCST. One additional participant was excluded because of completing less than half of the cWCST, resulting in a final sample of *N* = 34 (16 female) HC participants. Table A1 gives information about demographic and psychological data of HC participants. The median time between the two sessions for HC participants was 4 days (range: 1–16).

The two testing sessions were scheduled at the same time of the day, with the exception of two HC participants and three PD patients, whose day times between testing sessions deviated for more than 4 h. All participants in this study scored 21 or higher on the Montreal Cognitive Assessment [67]. All participants were offered a compensation of €25 per testing session. The study was approved by the ethics committee of Hannover Medical School (vote number: 6589). All participants gave informed consent in accordance with the Declaration of Helsinki. For further details, see [48,66].

### 2.3. Computerized Wisconsin Card Sorting Test

Participants were required to match stimulus cards to one of four key cards *W* = {one red triangle, two green stars, three yellow crosses, and four blue balls} according to one of three viable sorting categories *U* = {color, form, number} by pressing one of four keys *V* = {response 1, response 2, response 3, response 4}. Keys were spatially mapped to the position of key cards on the computer screen (see Figure 1). Stimulus cards varied on the three dimensions color, shape, and number but never shared more than one attribute with any of the key cards. The target display presented a stimulus card and key cards, which appeared invariantly above the stimulus card. Target displays remained on screen until a response was detected. Feedback cues were presented 800 ms after response detection and remained on screen for another 400 ms. The German words for repeat (i.e., ‘bleiben’) and shift (i.e., ‘wechseln’) served as positive and negative feedback cues, respectively. Feedback cues indicated whether the previous response was correct or incorrect and whether the applied sorting category should be shifted or repeated on the upcoming trial [68]. The next target display appeared 800 ms after feedback-cue offset. The correct sorting category switched randomly [69] after a minimum of two or more correct category repetitions. The average number of correct card sorts that was required to trigger a switch of the correct category was 3.5 trials. The cWCST was terminated after 39 switches of the correct sorting category or upon participant’s request. Prior to the experimental session, participants completed a short practice session including four category switches. Participants were explicitly informed about the viable sorting categories and about the fact that correct sorting categories change occasionally. The cWCST was programmed using Presentation^®^ and responses were collected on a Cedrus^®^ response pad.

### 2.4. Error Analysis

We analyzed perseveration errors (an erroneous repetition of the applied category following negative feedback) and set-loss errors (an erroneous switch of the applied category following positive feedback). We computed conditional error probabilities by dividing the number of committed errors by the number of trials on which a respective error type was possible (e.g., if six perseveration errors are committed on a total of 60 trials following negative feedback, the conditional perseveration error probability is 6/60 = 0.1). Conditional error probabilities were entered into Bayesian repeated measures analyses of variance (ANOVA) using JASP version 11.1 [70]. First, we analyzed the effect of PD pathology on conditional error probabilities by means of a Bayesian ANOVA including the within-subjects factor error type (perseveration error vs. set-loss error) and the between-subjects factor disease (HC vs. PD). For this analysis, we pooled individual conditional error probabilities across testing sessions. That is, we computed individual mean conditional error probabilities across the first and the second testing sessions. Second, we analyzed the effect of DA replacement therapy on conditional error probabilities of PD patients. We conducted a Bayesian ANOVA including the within-subjects factors error type (perseveration error vs. set-loss error) and medication (‘on’ vs. ‘off’ medication). For an analysis of effect of the testing session on conditional error probabilities, see Appendix B.

Results of Bayesian ANOVAs were reported as analysis of effects [71]. Evidence for an effect (i.e., a main effect of a factor or the interaction of factors) in the data was quantified by means of inclusion Bayes factors (BF_inclusion_). Inclusion Bayes factors gave the change from prior probability odds to posterior probability odds for the inclusion of an effect. Prior probabilities for the inclusion of an effect *p*(inclusion) were computed as the sum of prior probabilities of all ANOVA models which included the effect of interest. Likewise, posterior probabilities for the inclusion of an effect *p*(inclusion|data) were computed as the sum of all posterior probabilities of these ANOVA models. For example, the Bayesian ANOVA including the factors error type and disease considers five ANOVA models (i.e., a null model including no effects, two models including only the main effect of error type or the main effect of disease, a model including both main effects, and the full model including both main effects and the interaction effect of error type and disease). Each ANOVA model had a prior probability of 1/5 = 0.2. Thus, the prior probability for the inclusion of the main effect disease, which is included in three ANOVA models, is p(inclusion) = 3 × 0.2 = 0.6, giving a prior probability odd of 0.6/(1 − 0.6) = 1.5. The sum of posterior probabilities for the three ANOVA models including the main effect disease may be p(inclusion|data) = 0.9, giving a posterior probability odd of 0.9/(1 − 0.9) = 9. Following, the resulting inclusion Bayes factor is BF_inclusion_ = 9/1.5 = 6. For all Bayesian ANOVAs, default settings of JASP were used. We implemented uniform prior probabilities for all ANOVA models under consideration. For descriptive statistics, we reported mean conditional error probabilities together with 95% credibility intervals. 95% credibility intervals were computed as 1.96 standard errors of the mean around the mean.

### 2.5. Computational Modeling

For computational modeling of cWCST performance, we implemented the parallel RL model [44]. The parallel RL model conceptualizes cWCST performance as cognitive and sensorimotor learning, which occur in parallel, by means of Q-learning algorithms [53,54,72,73,74]. Q-learning algorithms operate on feedback prediction values, which quantify how strongly a positive or negative feedback is predicted following the application of a category or the execution of a response. Feedback prediction values are updated trial-wise in response to an observed feedback. The strength of the updating of feedback prediction values is modulated by prediction errors. Prediction errors are defined as the difference between the received feedback and the feedback prediction value. Higher prediction errors indicate stronger updating of feedback prediction values.

Individual parameters of the parallel RL model are learning rate parameters for cognitive and sensorimotor learning. Learning rates give the extent to which prediction errors are integrated into feedback prediction values of the applied category (for cognitive learning) or the executed response (for sensorimotor learning). In order to account for different strengths of learning from positive and negative feedback, learning rate parameters for cognitive and sensorimotor learning were further separated for trials following positive and negative feedback [61,73,75,76]. Highest values of learning rates indicate that a prediction error will be added to the feedback prediction value of the applied category or the executed response without attenuation. In contrast, with the lowest possible learning rate, no updating of the feedback prediction value of the applied category or the executed response will happen. The parallel RL model also incorporates cognitive and sensorimotor retention rates, which quantify how much information from previous trials will be retained for the current trial [64,77]. With highest values of retention rates, feedback prediction values from the previous trial will transfer to the current trial without mitigation. In contrast, with lowest values of retention rates, feedback prediction values are not transferred to the current trial. In such cases, responding depends entirely on the last received feedback. Lastly, an individual inverse temperature parameter gives the extent to which responding accords to integrated feedback prediction values. More precisely, the inverse temperature parameter indicates whether differences in integrated feedback prediction values are attenuated (inverse temperature values higher than 1) or emphasized (inverse temperature values less than 1). For a detailed description of the parallel RL model and further information about parameter estimation, see Appendix C.

We used Bayesian tests for direction to quantify evidence for effects of disease, medication, and session on model parameters [53,78]. In contrast to inclusion Bayes Factors, which we interpreted only if they were larger than 1, Bayes factors from Bayesian tests for direction were also interpreted if they were smaller than 1. As such, Bayes factors from Bayesian tests for direction indicate evidence for a decrease of a model parameter (BF < 1) or for an increase of a model parameter (BF > 1). For interpretation of evidential strength of Bayes factors, we followed [79]; Bayes factors larger than 3 (or less than 1/3) were interpreted as substantial evidence for the presence of an effect, Bayes factors larger than 10 (or less than 1/10) were interpreted as strong evidence for the presence of an effect, and Bayes factors larger than 100 (or less than 1/100) were interpreted as extreme evidence for the presence of an effect.

Inspection of individual conditional error probabilities and estimates of model parameters did not indicate any considerable effects of gender, time between testing sessions, and time of withdrawal from DA replacement therapy on conditional error probabilities and parameter estimates or on any comparisons involving these measures (see Appendix D). The implemented code can be downloaded from https://osf.io/nwrca, which also provides further specifications of hierarchical Bayesian analysis.

## 3. Results

### 3.1. Error Analysis

**Figure 2:**
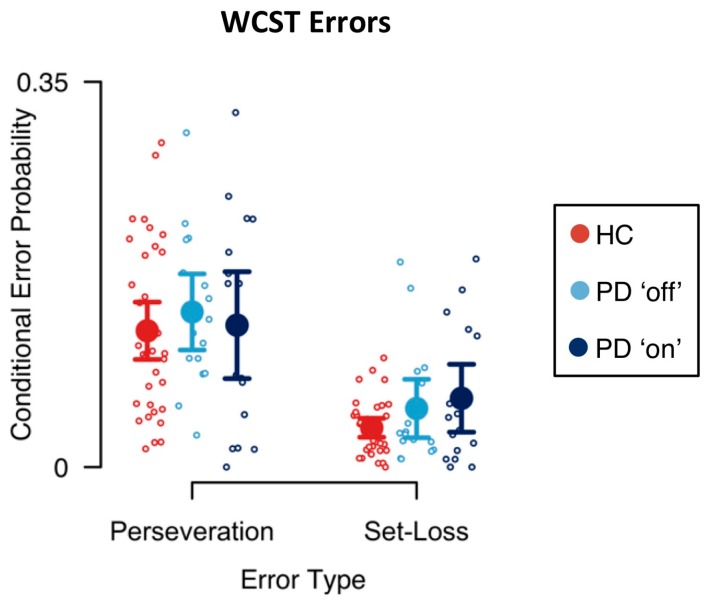
Conditional error probabilities. Large circles indicate mean conditional error probabilities. Error bars indicate the 95% credibility interval. Small circles indicate individual conditional error probabilities.

Mean conditional error probabilities are shown in Figure 2. First, we analyzed the effects of PD pathology on conditional error probabilities. Results of the Bayesian ANOVA including the between-subjects factor disease are reported in Table 1. There was extreme evidence for an effect of error type on conditional error probabilities (BF_inclusion_ > 1000). Conditional perseveration error probabilities were generally higher than conditional set-loss error probabilities. There was neither evidence for a main effect of disease on conditional error probabilities (BF_inclusion_ = 0.452) nor for the interaction effect of error type and disease on conditional error probabilities (BF_inclusion_ = 0.465).

Next, we analyzed the effects of DA replacement therapy in PD patients on conditional error probabilities. Results of the Bayesian ANOVA including the within-subjects factor medication are reported in Table 2. Again, we found extreme evidence for an effect of error type on conditional error probabilities (BF_inclusion_ > 1000). However, there was no evidence for a main effect of medication on conditional error probabilities (BF_inclusion_ = 0.247) and there was no evidence for an interaction effect of error type and medication on conditional error probabilities (BF_inclusion_ = 0.351).

### 3.2. Computational Modeling

**Figure 3:**
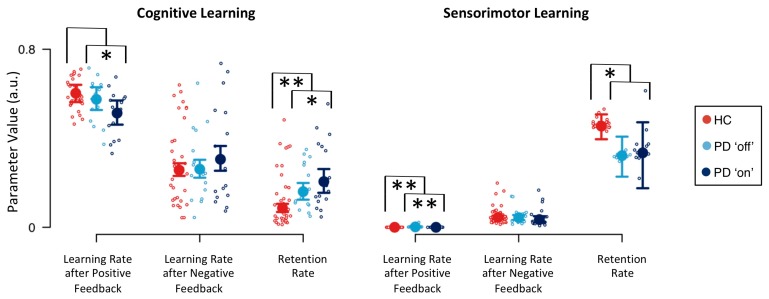
Model parameters for cognitive and sensorimotor learning. Large circles indicate medians of group-level posterior distributions (derived by Equation (A14)). Error bars indicate lower and upper quartiles of group-level posterior distributions. Small circles indicate medians of individual-level posterior distributions (derived by Equation (A13)); a.u. = arbitrary units; * substantial evidence for the presence of an effect; ** strong evidence for the presence of an effect.

Descriptive statistics of group-level model parameters for cognitive and sensorimotor learning are presented in Figure 3. Learning rate parameters were overall higher for cognitive learning than for sensorimotor learning, indicating a stronger impact of cognitive learning when compared to sensorimotor learning. Learning rate parameters for cognitive learning were higher after positive than after negative feedback. Thus, participants showed stronger cognitive learning from positive feedback than from negative feedback. In contrast, learning rate parameters for sensorimotor learning were smaller after positive feedback than after negative feedback. In fact, the sensorimotor learning rate after positive feedback was close to zero. Hence, participants showed sensorimotor learning from negative feedback, but they barely showed sensorimotor learning from positive feedback. The sensorimotor retention rate was higher than the cognitive retention rate. Participants retained more sensorimotor-learning information from previous trials than cognitive-learning information from previous trials. The inverse temperature parameter was less than 1 (HC: median = 0.150, Q_0.25_ = 0.142, Q_0.75_ = 0.158; PD ‘off’: median = 0.157, Q_0.25_ = 0.145, Q_0.75_ = 0.168; PD ‘on’: median = 0.152, Q_0.25_ = 0.138, Q_0.75_ = 0.165), indicating that differences in integrated feedback prediction values were emphasized.

Computational modeling analysis revealed three effects of PD pathology on model parameters: First, there was strong evidence for a decreased cognitive retention rate in HC participants (BF = 0.095; see Table 3). Second, there was substantial evidence for an increased sensorimotor retention rate in HC participants (BF = 4.725). PD patients retained more cognitive-learning information from previous trials when compared to HC participants. In contrast, PD patients retained less sensorimotor-learning information from previous trials than HC participants did. Third, there was strong evidence for a decrease of the sensorimotor learning rate parameter after positive feedback in HC participants (BF = 0.073). With regard to effects of DA replacement therapy in PD patients on model parameters, we found substantial evidence for a decreased cognitive learning rate after positive feedback for PD patients ‘on’ medication (BF = 0.282; see Table 3). Thus, PD patients ‘on’ medication showed reduced cognitive learning from positive feedback. This finding was mirrored by the sensorimotor learning rate after positive feedback, as there was strong evidence for a decrease of this model parameter when PD patients were tested ‘on’ medication (BF = 0.077). There was also substantial evidence for an increase of the cognitive retention rate when PD patients were tested ‘on’ medication (BF = 3.323). Thus, DA replacement therapy further increased the heightened cognitive retention rate of PD patients.

## 4. Discussion

The present data show how the construct of PD-related bradyphrenia [15,16,17,18,19,20,21,22,23], which is difficult to study with purely behavioral methods, can be investigated through applying computational techniques. Computational modeling of trial-by-trial reinforcement learning on the WCST [44] revealed that PD patients and HC participants learned similarly from trial-by-trial feedback on the cWCST. The two groups differed with regard to retention rates, with PD patients retaining (a) learned cognitive information from previous trials better, and (b) learned sensorimotor information from previous trials worse than HC participants did. We also found that systemic DA replacement therapy, which was titrated in individual patients for the best possible motility, incurred bradyphrenic side effects by (a) further increasing cognitive retention rates, and (b) decreasing cognitive learning from positive feedback. In the following sections, we discuss implications for neuropsychological sequelae of PD and DA replacement therapy, as well as implications for the investigation of brain–behavior relationships and for neuropsychological assessment. Furthermore, we outline study limitations and future research directions.

### 4.1. Implications for Neuropsychological Sequelae of PD

**Figure 4:**
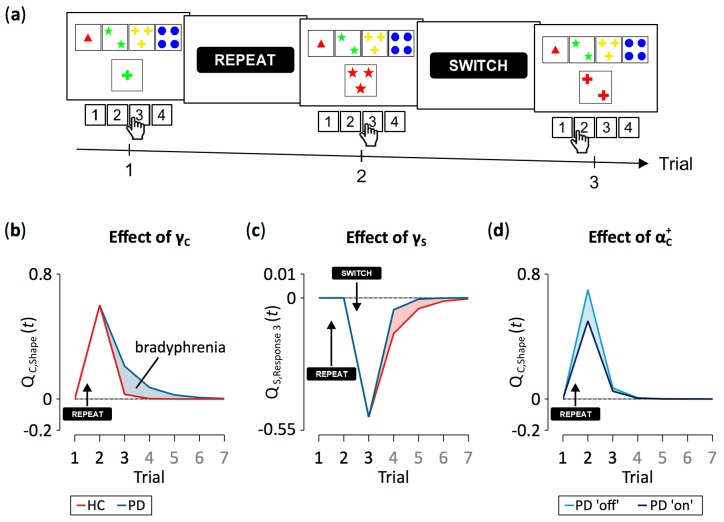
Exemplary effects of between-group variations of model parameters on trial-to-trial dynamics of feedback prediction values. (**a**) The representative trial sequence on the cWCST, as depicted in Figure 1. Panels (**b**), (**c**), and (**d**) give feedback prediction values across seven trials, the first three of them are shown in (**a**). Note that on Trials 4 to 7, neither the shape category was applied nor was response 3 pressed. Panel (**b**) shows cognitive-learning feedback prediction values for the application of the shape category. The received positive feedback on Trial 1 causes an increase in feedback prediction values. With high configurations of the cognitive retention rate (i.e., $\gamma_{C}$), such as seen in Parkinson’s Disease (PD) patients, more information from previous trials (i.e., feedback prediction values) was retained. Panel (**c**) shows sensorimotor-learning feedback prediction values for the execution of response 3. The execution of response 3 was followed by a positive feedback on Trial 1. However, as the sensorimotor learning rate for positive feedback was close to zero, no updating of feedback prediction values happened. On Trial 2, a negative feedback followed the execution of response 3, causing a decrease in feedback prediction values. With low values of the sensorimotor retention rate (i.e., $\gamma_{S}$ ), such as seen in PD patients, less sensorimotor-learning information from previous trials was retained. Panel (**d**) shows the effect of a low configuration of the cognitive learning rate for positive feedback (i.e., $\alpha_{C}^{+}$ ), such as seen in PD patients ‘on’ dopaminergic medication. Low learning rate configurations decrease learning from positive feedback, which is indicated by a reduced amplitude of feedback prediction values. All presented effects of model parameters on trial-to-trial dynamics of feedback prediction values were computed by varying exclusively the parameter of interest at arbitrary values while holding all other model parameters constant.

Computational modeling revealed that PD patients retained learned cognitive information from previous trials better than HC participants did (see Figure 3). Cognitive retention rates represent one of the latent variables in our computational RL model [44]. Higher cognitive retention rates indicate better retention (i.e., higher levels of activation) of objects of thought (i.e., categories in the cWCST context) through time. In this case, mental shifting between objects of thought will be hampered due to stronger proactive interference that is exerted from the retained categories. We conclude from our data that PD patients are characterized by a reduced flexibility of cognitive learning compared to HC participants (see Figure 4a,b for illustration). Thus, PD pathophysiology [2,3,4] is associated with a cognitive symptom, which can probably be best described as ‘inflexibility of thought’ (i.e., bradyphrenia; [19,20,21,22]). As a result, PD should not merely be considered as a ‘movement disorder’ because cognitive symptoms such as attentional inflexibility represent an integral manifestation of the disease around its mid-stage [2,3,4].

Traditional behavioral indicators of bradyphrenia are confounded by the presence of bradykinesia in PD [20]. For example, response times on more or less complex sensorimotor tasks represent a mixture of mental and motor slowing, and disentangling mental slowing from prolonged response times has proven difficult to achieve [20]. Computational modeling provides a technique for isolating less impure (latent) measures of bradyphrenia, which seem to be less contaminated by bradykinesia than response times are. Errors might likewise originate from a mixture of a variety of different mental processes, one of which is bradyphrenia. Our study provides a good example for the mixture problem: We found that cognitive retention rates, but not perseveration errors, were sensitive to group membership (see Figure 2 and Figure 3). Behaviorally manifested perseverative tendencies may occur as sequelae of bradyphrenia under certain circumstances, but they are the result of a variety of different mental processes, some of which may not be affected by the PD pathophysiology. As a result, manifest behavioral expressions of bradyphrenia seem to offer less-sensitive indicators of PD-related bradyphrenia than computationally derived latent variables do.

PD patients also retained learned sensorimotor information from previous trials worse than HC participants (see Figure 3). We defined sensorimotor learning as being concerned with response selection. Noticeable sensorimotor learning happened exclusively after the reception of negative feedback, indicating that participants tended to avoid responses that were followed by negative feedback. Reduced sensorimotor retention rates, such as that seen in PD patients when compared to HC participants, show that learned sensorimotor information (i.e., which response to avoid) dissipated more rapidly through time (see Figure 4a,c for illustration). These data reveal that PD pathophysiology [2,3,4,80] is associated with another mental symptom, which can probably be best described as impaired stimulus-response learning (or, in terms of sensorimotor learning, selecting a key card by executing a response). Impaired stimulus-response learning was repeatedly reported in PD patients, most frequently studied by means of probabilistic classification tasks [81,82,83].

### 4.2. Implications for Neuropsychological Sequelae of DA Replacement Therapy

We found that DA replacement therapy did not relieve PD-related cognitive symptoms. On the contrary, our results indicate that DA replacement therapy induces additional cognitive symptoms. That is, PD patients ‘on’ medication showed increased cognitive retention rates, indicating worsening of the already bradyphrenic attentional inflexibility compared to PD patients tested ‘off’ medication. In addition, PD patients ‘on’ medication showed decreased cognitive learning from positive feedback (see Figure 3). Reduced cognitive learning from positive feedback indicates that the activation of objects of thought (i.e., categories) achieves attenuated peaks, thereby inducing attentional instability (see Figure 4a,d for illustration). Thus, our results are in line with previous research, demonstrating that DA replacement therapy may induce iatrogenic cognitive impairments [1,5,7,8,10,11,12].

It is well-recognized that performance on cognitive tasks depends on optimal levels of DA, implying that both an insufficient and an excessive level of DA impairs performance on such tasks [1,5,7,8,10,11,12]. In early PD, DA depletion is most severe in the dorsal striatum, which is part of the nigro-striatal DA system. Other DA systems appear to be relatively spared from DA depletion in early PD, such as the meso-limbic and the meso-cortical DA systems [10]. DA replacement therapy of PD is titrated to ameliorate motor symptoms, aiming to restore the missing DA in the dorsal striatum. An optimal DA replacement therapy may relieve motor symptoms, but it can cause an overdosing of the less DA-depleted meso-limbic and meso-cortical DA systems. Conclusively, the cognitive impairments that were induced by DA replacement therapy (see above) in this study might occur as a corollary of excessive DA levels in the meso-limbic and/or the meso-cortical DA system [1,5,7,8].

This study found that DA replacement therapy incurred two iatrogenic side effects, i.e., attentional inflexibility, indicated by increased cognitive retention rates, and attentional instability, indicated by decreased cognitive learning from positive feedback. The meso-cortical DA system plays a crucial role in attentional flexibility [84,85,86], whereas the meso-limbic DA system is associated with anticipation of reward or positive feedback [87,88]. Thus, the iatrogenic cognitive impairments induced by DA replacement therapy could possibly be subserved by distinct DA systems [89]; the overstimulation of the meso-cortical DA system might cause attentional inflexibility, as indicated by an increased cognitive retention rate, whereas an overstimulation of the meso-limbic DA system might induce attentional instability via reduced cognitive learning from positive feedback.

### 4.3. Implications for Brain–Behavior Relationships

Computational models specify explicit cognitive architectures, i.e., cognitive components and their exactly defined ways of interaction. We considered interactions between explicitly defined reinforcement learning processes (Equations (A2), (A3), (A6) and (A7)), plus simple mechanisms of short-term retention (Equations (A1) and (A5)), as the cognitive architecture, which was finally expressed as overt behavior on the cWCST. The particular cognitive architecture of our computational model may be best described as parallel RL, and the question how well competing models fit behavioral cWCST data has been addressed elsewhere [44]. The results from that study led to the conclusion that parallel RL provides a better conceptualization of behavioral cWCST data than competing models do, thereby lending initial credibility to the potential adequacy of parallel RL as a suitable descriptor of some of the covert cognitive processes that underlie overt behavior on the WCST.

The issue of how computationally derived cognitive processes are mapped to neural mechanisms deserves further inquiry. In particular, details of the underlying neural mechanisms that are associated with the latent variables that can be gained from applying our parallel RL model, such as individual learning rate or retention rate parameters or trial-wise feedback prediction values or prediction errors, should be addressed by appropriate brain imaging studies. The approach to combine computational modeling and brain imaging represents a promising avenue for the advancement of brain–behavior relationships [49,53]. The main reason why we consider this approach as a promising technique is that computationally derived latent variables may provide less impure indicators of covert cognitive processes than behavioral indicators do. As such, the parallel RL model conceptualizes the final behavioral outcome on any trial of the cWCST as emerging from a mixture of many interacting, but isolable, componential processes (Equations (A8) and (A9)).

### 4.4. Implications for Neuropsychological Assessment

To date, clinical neuropsychological assessment of cognitive dysfunctions relies almost exclusively on the results that can be obtained from behavioral observations. The WCST is just one of the many examples of how clinical neuropsychological assessment usually works: Test authors and examiners draw ad-hoc conclusions about cognitive dysfunctions, which are based on counts of the occurrence of particular behavioral events, such as—in the example of the WCST—the number of perseveration and/or set-loss errors committed. Clinical neuropsychological assessment refers to cognitive assessment, albeit these covert cognitive processes remain unobservable. Thus, clinical neuropsychological assessment involves inferences that clearly exceed the behavioral observations. With regard to the WCST, typical inferences would be that a patient who showed corresponding behavioral signs suffers from impaired abstraction, or from cognitive inflexibility and/or distractibility. Cognitive constructs utilized for clinical neuropsychological assessment are often ill-defined, bearing the problem that they appear as arbitrary re-descriptions of the behavioral observations that were made.

Computational modeling bears the potential for clinical neuropsychological assessment to improve its inferential capability. Specifically, latent variables that represent computationally derived reflections of presumed cognitive processes may replace the traditional verbal constructs of clinical neuropsychological assessment. As noted above, behavioral indicators are better conceived as resulting from a mixture of many contributing covert processes. Selecting just one ‘main’ process may constitute an over-simplified inference. Computationally derived latent variables may be less susceptible to this shortcoming of the traditional manner in which clinical neuropsychological assessment is practiced. In that regard, it should be noted that our parallel RL model yields—for each individual—a set of latent variables. We found that some of these latent variables, but not directly observed behavioral indicators (i.e., conditional error probabilities; see Figure 2), were sensitive to disease/medication status. This sensitivity gradient may occur because the latent variables are less impure than behavioral indicators are. We also showed that the variability of the latent variables is unique, i.e., that it is non-redundant with regard to the conventional measures that are available from non-computational investigation (Table A7). However, it remains a possibility that decomposing observable behavioral indicators into less impure error scores provides another route towards an assessment of more specific cognitive dysfunctions (e.g., [27,79,90]). However, until now, this route has not led to sufficiently pure behavioral measures of cognitive dysfunctions.

There are some relatively straightforward clinical implications of computational cognitive neuropsychology. Considering the present study as an exemplary forerunner of that approach, the pursuit of computational cognitive neuropsychology may change our way of caring for patients with chronic neurological diseases such as PD in several ways. First, our general diagnostic work-up may change as outlined in the previous paragraphs, shifting our focus away from behavioral observations toward latent cognitive variables. Latent variables may provide more detailed and less impure information about cognitive sequelae of chronic neurological diseases and their progression. This could enable us to trace, and hopefully predict the long-term course of individual patients. Second, given that some of the latent cognitive variables were sensitive to adverse effects of DA treatment, such computational diagnostic work-ups may guide titrating DA medication dosage in individual PD patients in such a way that desired treatment effects are at their optimum while non-desirable treatment effects are minimized.

### 4.5. Study Limitations and Directions for Future Research

PD was shown to be robustly associated with impaired behavioral WCST indicators (for a meta-analysis, see [28]). In this study, mean conditional set-loss error probabilities—and to a lesser extent conditional perseveration error probabilities—were increased in PD patients when compared to HC participants (see Figure 2). However, we did not find evidence for an effect of disease on conditional error probabilities (see Table 1), most likely due to the relatively small sample size, which constitutes a limitation of this study. Thus, a replication study with larger sample size might reveal more nuanced effects of PD pathophysiology and/or DA replacement therapy. Furthermore, results of Bayesian repeated measures ANOVAs should be interpreted with caution, since conditional error probabilities appeared to be skewed towards zero, which might indicate a violation of the assumption of normally distributed conditional error probabilities.

Furthermore, DA replacement therapy was not fully balanced across first and second testing sessions, which might limit the interpretability of our results. However, in computational modeling analysis, we countered this problem by explicitly accounting for the repeated cWCST administration, allowing us to disentangle effects of DA replacement therapy from session effects on model parameters (see Appendix E). However, our results need to be consolidated using a fully balanced study design. Furthermore, effects of DA medication, particularly L-Dopa, can last for several days after withdrawal [91]. Thus, the time of withdrawal from DA replacement therapy in many PD patients of this study (median = 14 h) might have been too short for a complete DA depletion. L-Dopa and dopamine agonists were shown to have differentiable cognitive effects [5]. However, the majority of PD patients in this study received a mixture of L-Dopa and DA agonists (see Table A2). Hence, cognitive effects of L-Dopa and DA agonists were not dissociable in this study; their specific effects should be addressed by future research. When investigating the cognitive effects of DA medication, it is also important to consider the DA receptors that are targeted by the administered DA medication [11,92], as DA receptor subtypes may be related to distinct cognitive processes [61,73,93,94].

Results of the Montreal Cognitive Assessment did not indicate that any PD patient was affected by mild cognitive impairment (MCI) [95]. However, the Montreal Cognitive Assessment represents a fast assessment tool for screening potential MCI in PD. Thus, a reliable diagnosis based on a comprehensive assessment is missing [96], which constitutes a further limitation of this study.

In our earlier study, we concluded that parallel RL provides a better conceptualization of behavioral cWCST data than competing models do [44]. It remains an open question whether this conclusion, which was based on the analysis of a large sample of young volunteers (*N* = 375), is generalizable to other populations, such as PD patients. In this study, we did not consider competing computational models because the relatively small sample size limited the validity of such model comparison efforts. Future studies should—whenever appropriate—consider all candidate computational models and base further analyses on the computational model that provides the best conceptualization of behavioral data [97].

Estimates of the sensorimotor learning rate following positive feedback were close to zero (see Figure 3), which is in line with results of our previous study [44]. Thus, the sensorimotor learning did virtually not happen following positive feedback (see Figure 4a,d for illustration; for a detailed discussion, see [44]). As even highest estimates of the sensorimotor learning rate following positive feedback were negligible in size and variance when compared to estimates of other learning rate parameters, we refrained from any interpretation of disease and medication effects on this close-to-zero model parameter.

Our parallel RL model provides a route towards the advanced assessment of cognitive dysfunctions. In this exemplary study, we analyzed data obtained from a computerized variant of the WCST, i.e., the cWCST. However, the parallel RL is not restricted to analysis of cWCST data but it is rather applicable to data obtained by any available WCST variant [41,98,99,100]. The sole requirement for implementing the parallel RL model is that data must be provided in a trial-by-trial format, rendering our computational modeling approach a promising method for (re)analyzing clinical WCST data. In order to increase the power of computational modeling analysis, such data sets might be merged across several studies and/or research centers.

There are two major statistical advantages of the implemented computational approach over the conducted error analysis. First, we estimated model parameters under consideration of individual trial-by-trial dynamics (as defined by the parallel-RL model), whereas conditional error probabilities were computed by simply aggregating the number of committed perseveration or set-loss errors. Second, we used hierarchical Bayesian analysis for parameter estimation [74,101]. Hierarchical Bayesian analysis provides high statistical power by considering individual differences in model parameters while pooling information across all individuals by means of group-level parameters. In contrast, group-level statistics of error analysis were computed as mean conditional error probabilities.

## 5. Conclusions

PD patients showed (a) bradyphrenic attentional inflexibility, and (b) reduced sensorimotor retention. DA medication does not remediate any of these cognitive symptoms. On the contrary, DA medication decreases cognitive learning from positive feedback in PD patients, thereby inducing attentional instability. We discussed the iatrogenic effects of DA medication as probably originating from overdosing meso-limbic/cortical DA systems. In conclusion, apart from reduced sensorimotor retention, PD patients who are under DA medication are prone to (a) bradyphrenic attentional inflexibility, probably due to the characteristic brain pathophysiology, and (b) attentional instability, probably due to iatrogenic effects of DA medication. These insights were made possible through the application of a computational RL model [44], which served to decompose WCST performance into covert constituent parts and provided latent variables that may be less impure than behavioral indicators of cognitive impairments. These computationally derived latent variables should be utilized in future research for investigating brain–behavior relationships. The latent variables may also be utilized to derive novel neuropsychological assessment techniques for bradyphrenia/attentional flexibility in PD and in other neurological diseases and psychiatric disorders.

## Figures and Tables

**Table 1:** Analysis of effects of error type and disease on conditional error probabilities.

Effects	*p*(Inclusion)	*p*(Inclusion|Data)	BF_inclusion_
Error Type	0.600	>0.999	>1000 ***
Disease	0.600	0.404	0.452
Error Type x Disease	0.200	0.104	0.465

*** extreme evidence.

**Table 2:** Analysis of effects of error type and medication on conditional error probabilities.

Effects	*p*(Inclusion)	*p*(Iinclusion|Data)	BF_inclusion_
Error Type	0.600	>0.999	>1000 ***
Medication	0.600	0.270	0.247
Error Type x Medication	0.200	0.081	0.351

*** extreme evidence.

**Table 3:** Bayes factors for effects of disease and medication on model parameters.

Parameter	Definition	Effect	
		Disease	Medication
$\alpha_{C}^{+}$	cognitive learning rate following positive feedback	1.519	0.282 *
$\alpha_{C}^{-}$	cognitive learning rate following negative feedback	0.940	2.676
$\gamma_{C}$	cognitive retention rate	0.095 **	3.323 *
$\alpha_{S}^{+}$	sensorimotor learning rate following positive feedback	0.073 **	0.077 **
$\alpha_{S}^{-}$	sensorimotor learning rate following negative feedback	1.137	0.521
$\gamma_{S}$	sensorimotor retention rate	4.725 *	1.075
τ	inverse temperature parameter	0.551	0.720

* substantial evidence; ** strong evidence.

**Figure A1:**
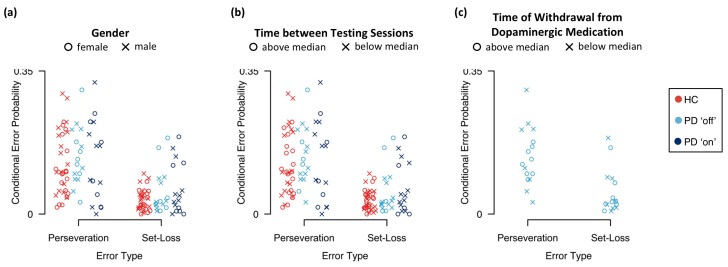
Individual conditional error probabilities separated by (**a**) gender, (**b**) median time between testing sessions, and (**c**) median time of withdrawal from dopaminergic medication.

**Figure A2:**
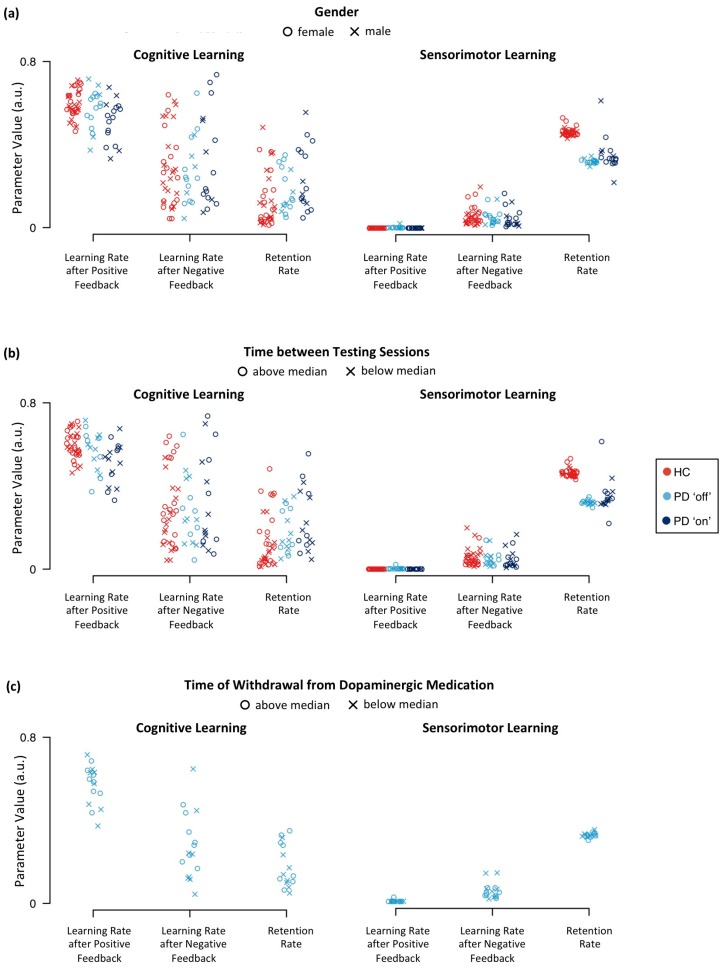
Medians of individual-level posterior distributions (derived by Equation (A13)) separated by (**a**) gender, (**b**) median time between testing sessions, and (**c**) median time of withdrawal from dopaminergic medication.

## Appendix A

**Table A1:** Demographic, clinical, and psychological characteristics.

	Healthy Control Participants (*N* = 34)			Parkinson’s Disease Patients (*N* = 16)		
	Mean	*SD*	*n*	Mean	*SD*	*n*
Age (years)	63.18	9.71	34	59.94	8.84	16
Education (years)	13.49	4.09	34	14.40	2.88	15
Disease duration (years)	-	-	-	6.75	4.04	16
UPDRS III ‘on’	-	-	-	17.75	8.05	16
UPDRS III ‘off’	-	-	-	27.92	12.68	13
LEDD	-	-	-	932.63	437.86	16
MoCA (cognitive status)	28.35	1.97	34	27.47	1.08	16
WST (premorbid intelligence)	30.71	3.79	34	23.00	32.71	16
AES (apathy)	10.77	6.81	34	15.31	8.85	16
BDI-II (depression)	6.59	8.28	34	6.56	4.43	16
BSI-18 (psychiatric status)	6.16	7.99	33	7.07	4.89	14
Anxiety	1.82	2.31	33	2.79	1.37	14
Depression	1.91	3.14	33	1.29	1.33	14
Somatization	2.44	3.26	33	3.00	2.96	14
SF-36 (health status)	74.21	20.12	33	59.07	16.66	14
Physical functioning	79.55	22.65	33	56.79	24.15	14
Physical role functioning	71.97	40.87	33	35.71	41.27	14
Bodily pain	71.58	26.20	33	61.71	26.67	14
General health perception	60.33	18.35	33	51.29	20.08	14
Vitality	63.79	19.61	33	54.29	17.85	14
Social role functioning	88.26	16.52	33	65.18	24.60	14
Emotional role functioning	84.91	30.11	33	83.36	36.39	14
Mental health	77.58	15.84	33	67.71	12.10	14
BIS-brief (impulsiveness)	15.45	4.17	34	14.84	3.74	16
DII (impulsivity)						
Functional	5.85	2.95	34	6.00	2.45	16
Dysfunctional	2.38	2.59	34	3.31	3.95	16
QUIP-RS (impulse control)	0.61	1.42	28	6.00	9.78	15
SPQ (schizotypal traits)	4.46	3.67	33	4.57	3.67	14
Interpersonal	2.52	2.41	33	2.36	1.91	14
Cognitive-perceptual	1.15	1.18	33	1.07	1.39	14
Disorganized	0.79	1.11	33	1.14	1.51	14

Disease duration was defined as the difference between the year of testing and the year of Parkinson’s disease symptom onset in the medical records; UPDRS III = Unified Parkinson’s Disease Rating Scale-part III; LEDD = Levodopa Equivalent Daily Dosage (mg per day) [102]; MoCA = Montreal Cognitive Assessment [67]; WST = Wortschatztest [103]; AES = Apathy Evaluation Scale [104]; BDI-II = Beck Depression Inventory-II [105]; BSI- 18 = Brief Symptom Inventory (18-item version) [106]; SF-36 = Short Form Health Survey [107]; BIS-Brief = Barratt Impulsiveness Scale (8-item version) [108]; DII = Dickman Impulsivity Inventory [109]; QUIP-RS = Questionnaire for Impulsive-Compulsive Disorders in Parkinson’s Disease—Rating Scale [110]; SPQ = Schizotypal Personality Questionnaire [111].

**Table A2:** Parkinson’s disease patients’ medication and LEDD scores.

Patient Number	Medication (mg)	LEDD
1	Pramipexole 3.15	450
2	Pramipexole 0.52, Rasagiline 1	175
3	Pramipexole 3.15, L-Dopa 300, C-L-Dopa 100	825
4	L-Dopa 400, Amantadine 200, Rasagiline 1, Rotigotine 12	1060
5	L-Dopa 1000, Entecapone 1000, Pramipexole 1.75, Oral Selegiline 10	1680
6	L-Dopa 400	400
7	L-Dopa 600, Entecapone 600, Pramipexole 1.04	948
8	L-Dopa 350, C-L-Dopa 500, Pramipexole 2.1	1025
9	L-Dopa 400, Rotigotine 2	460
10	L-Dopa 550, Rotigotine 16, Rasagiline 1, Amantadine 200	1330
11	L-Dopa 600, Amantadine 200, Rotigotine 6, Cabergoline 6	1380
12	L-Dopa 600, C-L-Dopa 100, Entecapone 1200, Pramipexole 1.57	1098
13	L-Dopa 600, C-L-Dopa 300, Entecapone 800, Cabergoline 6	1497
14	L-Dopa 700, C-L-Dopa 100, Entecapone 600, Rotigotine 8	1246
15	L-Dopa 500, Piribidil 50	550
16	L-Dopa 600, Entecapone 800	798

LEDD = Levodopa Equivalent Daily Dosage (mg per day) [102]; C-L-Dopa = Controlled-Release L-Dopa; Pramipexole was reported as base form dosis. For computation of LEDD scores, the salt form was used (Pramipexole dihydrochloride 1 H_2_O) [102].

## Appendix B

Descriptive statistics of conditional error probabilities for the first and second testing sessions are reported in Table A3 Bayesian ANOVA with the factors error type (perseveration error vs. set-loss error) and session (first vs. second session; see Table A4) revealed extreme evidence for an effect of error type on conditional error probabilities (BF_inclusion_ > 1000). There was also extreme evidence for a main effect of session on conditional error probabilities (BF_inclusion_ = 319.214), indicating that conditional error probabilities were generally reduced on the second testing session. There was no evidence for the interaction effect of error type and session on conditional error probabilities (BF_inclusion_ = 1.962).

**Table A3:** Descriptive statistics of conditional error probabilities for first and second testing sessions (mean with 95% credibility interval in parentheses).

Error Type	Session	
	First	Second
Perseveration Error	0.145 (0.121, 0.169)	0.110 (0.086, 0.133)
Set-Loss Error	0.052 (0.039, 0.064)	0.034 (0.023, 0.044)

**Table A4:** Analysis of session effects on conditional error probabilities.

Effects	*p*(Inclusion)	*p*(Inclusion|Data)	BF_inclusion_
Error Type	0.600	>0.999	>1000 ***
Session	0.600	0.998	319.214 ***
Error Type x Session	0.200	0.329	1.962

*** extreme evidence.

## Appendix C

The cognitive Q-learning algorithm operates on feedback prediction values of sorting categories. More precisely, $Q_{C}\left(t\right)$ is a 3 (categories) × 1 vector that gives feedback prediction values for the application of any category on trial *t*. For trial-wise updating of feedback prediction values, the strength of transfer of feedback prediction values from trial *t* to trial *t*+1 is modeled as:

(A1) $$Q_{C}^{\prime }\left(t\right)=\gamma_{C}{*Q}_{C}\left(t\right)$$

where $\gamma_{C}$ ϵ [0,1] is an individual cognitive retention rate. High values of $\gamma_{C}$ represent a strong impact of previous feedback prediction values on current responding. Next, trial-wise prediction errors $\delta_{C}\left(t\right)$ are computed with regard to the applied category *u* ϵ *U* on trial *t* as:

(A2) $$\delta_{C}\left(t\right)=r\left(t\right)-Q_{C,u}^{\prime }\left(t\right)$$

where *r*(*t*) is 1 or −1 for a received positive or negative feedback, respectively. Feedback prediction values for the application of categories are updated following a delta-learning rule:

(A3) $$Q_{C}\left(t+1\right)=Q_{C}^{\prime }\left(t\right)+Y_{C}\left(t\right)*\alpha_{C}*\delta_{C}\left(t\right)$$

$Y_{C}\left(t\right)$ is a 3 × 1 dummy vector, which is 1 for the applied category *u* and 0 for all other categories on trial *t*. $Y_{C}\left(t\right)$ ensures that only the feedback prediction values for the applied category *u* on trial *t* is updated by the prediction error $\delta_{C}\left(t\right)$. Independent individual learning rate parameters for positive and negative feedback, $\alpha_{C}^{+}$ ϵ [0,1] and $\alpha_{C}^{-}$ ϵ [0,1], respectively, quantify the strength of learning from the presence of a prediction error. Feedback prediction values for the application of categories $Q_{C}\left(t+1\right)$ are matched to responses, which is represented by a 4 (responses) × 1 vector $Q_{RC}\left(t+1\right)$. For response *v* ϵ *V* on trial *t*+1, $Q_{RC,v}\left(t+1\right)$ is computed as:

(A4) $$Q_{RC,v}\left(t+1\right)=X_{v}^{T}\left(t+1\right){\;Q}_{C}\left(t+1\right)$$

with $X_{v}\left(t+1\right)$ is a 3 (categories) × 1 vector that represents the match between a stimulus card and response *v* with regard to sorting categories. Let $X_{v}\left(t+1\right)$ be 1 if a stimulus card matches response *v* by category *u* and let $X_{v}\left(t+1\right)$ be 0 elsewise. $X_{v}^{T}\left(t+1\right)$ denotes the transpose of $X_{v}\left(t+1\right)$. To account for responses that match no viable sorting category (i.e., responses that certainly yield a negative feedback), feedback prediction values of such responses are assigned a value of $Q_{RC,v}\left(t+1\right)=-1$.

Sensorimotor Q-learning parallels cognitive Q-learning. However, sensorimotor Q-learning directly operates on feedback prediction values for the execution of responses. Hence, a 4 (responses) × 1 vector $Q_{S}\left(t\right)$ gives feedback prediction values for the execution of any response on trial *t*. Again, the strength of the transfer of feedback prediction values from trial *t* to trial *t*+1 is modeled as:

(A5) $$Q_{S}^{\prime }\left(t\right)=\gamma_{S}{*Q}_{S}\left(t\right)$$

with the individual sensorimotor retention rate $\gamma_{S}$ ϵ (0,1). Trial-wise prediction errors $\delta_{S}\left(t\right)$ with regard to the executed response *v* on trial *t* are computed as:

(A6) $$\delta_{S}\left(t\right)=r\left(t\right)-Q_{S,v}^{\prime }\left(t\right)$$

Feedback prediction values for the execution of responses are then updated as:

(A7) $$Q_{S}\left(t+1\right)=Q_{S}^{\prime }\left(t\right)+Y_{S}\left(t\right)*\alpha_{S}*\delta_{S}\left(t\right)$$

Again, $Y_{S}\left(t\right)$ is a 4 × 1 dummy vector that is 1 for the executed response *v* and 0 for all other responses on trial *t*, which ensured that only feedback prediction values of the executed response are updated by the prediction error. For sensorimotor learning, we assumed independent learning rate parameters for positive and negative feedback, $\alpha_{S}^{+}$ ϵ [0,1] and $\alpha_{S}^{-}$ ϵ [0,1], respectively.

Response probabilities are computed by linear integrating feedback prediction values of cognitive and sensorimotor Q-learning. Integrated feedback prediction values $Q_{sum}\left(t+1\right)$ on trial *t*+1 are computed as:

(A8) $$Q_{sum}\left(t+1\right)=Q_{RC}\left(t+1\right)+Q_{S}\left(t+1\right)$$

The probability of executing response *v* on trial *t*+1 is computed using a “softmax” logistic function on integrated feedback prediction values as:

(A9) $$P_{v}\left(t+1\right)=\frac{e^{\frac{Q_{sum,v}\left(t+1\right)}{\tau }}}{\sum_{j=1}^{4}e^{\frac{Q_{sum,j}\left(t+1\right)}{\tau }}}$$

with τ∈[0, ∞] is an individual inverse temperature parameter, indicating whether differences in integrated feedback prediction values are attenuated (*τ* > 1) or emphasized (0 < *τ* < 1).

Parameter estimation of the parallel RL model was carried out by means of hierarchical Bayesian analysis [74,76,101,112,113,114,115,116] using RStan [117]. Hierarchical Bayesian analysis assumes that individual-level model parameters are nested within group-level model parameters, which allows parameter estimation with high statistical power [65,101,112]. More precisely, the parallel RL model comprised seven model parameters $Z\in \left[\alpha_{C}^{+},\alpha_{C}^{-},\gamma_{C},\alpha_{S}^{+},\alpha_{S}^{-},\gamma_{S},\tau \right]$. Each individual-level model parameter was drawn from a group-level normal distribution with mean $\mu_{z}$ and standard deviation $\sigma_{z}$. In order to facilitate hierarchical Bayesian analysis, we used a non-centered parameterization [74,118]. That is, we sampled individual-level model parameters from a standard normal distribution that was multiplied by $\sigma_{z}$ (i.e., the group-level scale parameter) and shifted by $\mu_{z}$ (i.e., the group-level location parameter). Individual deviation from $\mu_{z}$ of participant *i* was introduced by location parameter ${z^{\prime }}_{i}$. Thus, model parameter $z_{i}$ was parameterized as:

(A10) $$z_{i}=\mu_{z}+\sigma_{z}*{z^{\prime }}_{i}$$

In order to research the relationships of PD pathology and DA replacement therapy with model parameters, we extended hierarchical Bayesian analysis following the procedure outlined by [119] (see also [53]). First, to account for associations of PD pathology with model parameters, we introduced the between-subjects effect disease. Disease-related shifts of model parameters were accounted for by adding parameter $\mu_{disease,z}$ to the group-level location parameter $\mu_{Z}$ exclusively for HC participants. Second, in order to account for medication-related shifts of model parameters, we introduced the within-subjects effect medication. For all testing sessions of PD patients ‘on’ medication, we added the variable $medication_{z,i}$ to the group-level location parameter $\mu_{z}$. As medication was a within-subjects effect, it was a participant-specific random variable that was drawn from a group-level normal distribution with location parameter $\mu_{medication,z}$, standard deviation $\sigma_{medication,z}$ and individual-level location parameters ${z^{\prime }}_{medication,i}$. Again, we used a non-centered parameterization for $medication_{z,i}$ as:

(A11) $$medication_{z,i}=\mu_{medication,z}+\sigma_{medication,z}*{z^{\prime }}_{medication,i}$$

In order to account for variance in model parameters that was introduced by the repeated administration of the cWCST, we introduced the within-subjects effect session. The variable $session_{z,i}$ was added to the group-level location parameter $\mu_{z}$ for all second testing sessions. $session_{z,i}$ was again a participant-specific random variable that was drawn from a group-level normal distribution with location parameter $\mu_{session,z}$, standard deviation $\sigma_{session,z}$, and individual-level location parameter ${z^{\prime }}_{session,i}$:

(A12) $$session_{z,i}=\mu_{session,z}+\sigma_{session,z}*{z^{\prime }}_{session,i}$$

Note that session effects were not of primary interest for this study. For analyses of session effects, see Appendix E.

To further facilitate hierarchical Bayesian analysis, we conducted parameter estimation in an unconstrained space [74,76,118], i.e., location and scale parameters were free to vary without constraints. As model parameters had upper and lower boundaries, the linear integration of location and scale parameters (Equation (A13)) was mapped to the interval [0, 1] using the Probit function. The Probit is the inverse-cumulative standard normal distribution. Note that this procedure is also appropriate for the inverse temperature parameter, which did not exceed estimates of 1 [44].

In summary, hierarchical Bayesian analysis included data from *J* = 100 cWCST administrations that were obtained from 34 HC participants and 16 PD patients, who were all administered the cWCST twice. For PD patients, testing sessions were conducted either ‘on’ or ‘off’ dopaminergic medication. Thus, model parameter $z_{j}$ of cWCST administration *j* was defined as:

(A13) $$z_{j}=Probit\left(\mu_{z}+\sigma_{z}*{z^{\prime }}_{i}+I_{d,j}*\mu_{disease,z}+I_{m,j}*medication_{z,i}+I_{s,j}*session_{z,i}\right)$$

In order to account for the potential role of disease, medication, and session in cWCST administration *j*, we included a set of dummy variables I. Disease-related shifts of model parameters were modeled by including parameter $\mu_{disease,z}$ exclusively for HC participants. Thus, $I_{d,j}$ was coded 1 if cWCST administration *j* was obtained from a HC participant and 0 otherwise. Shifts of model parameters for cWCST administrations ‘on’ medication were modeled by variable $medication_{z,i}$. Thus, $I_{m,j}$ was coded 1 if cWCST administration *j* was obtained from a PD patient ‘on’ medication and 0 otherwise. The variable $session_{z,i}$ was exclusively added for second testing sessions. Thus, $I_{s,j}$ was coded 1 if cWCST administration *j* was a second testing sessions and 0 otherwise.

In line with previous studies [44,74], all location parameters had normal prior distributions (*μ* = 0, *σ* = 1) and all scale parameters had half Cauchy prior distributions (*μ* = 0, *σ* = 5). Sampling was done using three chains of 1000 iterations, including 500 warm-up iterations per chain. Convergence of chains was checked visually by trace-plots and quantitatively by the $\hat{R}$ statistic [120].

Bayes factors (BF) for effects of disease, medication, and session were computed by dividing the posterior density of parameters $\mu_{disease,z}$, $\mu_{medication,z}$, or $\mu_{session,z}$ above zero by the respective posterior density below zero. This method is appropriate as prior distributions of $\mu_{disease,z}$, $\mu_{medication,z}$, and $\mu_{session,z}$ were symmetric around zero [121].

For descriptive statistics of model parameters, we analyzed posterior distributions of group-level location parameters. The group-level posterior distribution of model parameter *z* was computed as:

(A14) $$z=Probit\left(\mu_{z}+I_{d}*\mu_{disease,z}+I_{m}*\mu_{medication,z}+I_{s}*\mu_{session,z}\right)$$

Dummy variables *I* were used to compute group-level model parameter posterior distributions for HC participants ($I_{d}=1,\;I_{m}=0$) and PD patients ‘off’ ($I_{d}=0,\;I_{m}=0$) and ‘on’ ($I_{d}=1,\;I_{m}=1$) medication. As we were not interested in session effects on model parameters, we averaged posterior distributions of model parameters across estimates of first ($I_{s}=0)$ and second testing sessions ($I_{s}=1)$.

## Appendix E

For descriptive statistics of model parameters for the first and second testing session, we averaged posterior distributions of model parameters across HC participants and PD patients (Table A5).

**Table A5:** Descriptive statistics of group-level posterior distributions of model parameters for first and second testing sessions (median with lower and upper quartiles in parentheses).

Parameter	Session	
	First	Second
$\alpha_{C}^{+}$	0.517 (0.477, 0.558)	0.612 (0.573, 0.652)
$\alpha_{C}^{-}$	0.219 (0.191, 0.249)	0.342 (0.307, 0.378)
$\gamma_{C}$	0.121 (0.096, 0.148)	0.188 (0.154, 0.225)
$\alpha_{S}^{+}$	0.004 (0.002, 0.009)	<0.001 (<0.001, 0.001)
$\alpha_{S}^{-}$	0.054 (0.046, 0.065)	0.031 (0.024, 0.039)
$\gamma_{S}$	0.345 (0.274, 0.406)	0.401 (0.307, 0.480)
τ	0.149 (0.139, 0.158)	0.157 (0.148, 0.167)

There was strong and extreme evidence for an increase of cognitive learning rate parameters from the first to the second testing session (BF = 15.854 and BF = 1499.000 following positive and negative feedback, respectively; see Table A6). There was also strong evidence for a decrease of sensorimotor learning rate parameters from the first to the second testing session (BF = 0.046 and BF = 0.016 following positive and negative feedback, respectively). Cognitive learning from feedback was stronger on the second testing session when compared to the first testing session. In contrast, sensorimotor learning was less strong on the second testing session when compared to the first testing session. Additionally, there was strong evidence for an increase of the cognitive retention rate from the first to the second testing session (BF = 47.387), indicating that more information from previous trials retained in the cognitive on the second testing session.

**Table A6:** Bayes factors for effects of session on model parameters.

Parameter	Bayes Factor
$\alpha_{C}^{+}$	15.854 **
$\alpha_{C}^{-}$	1499.000 ***
$\gamma_{C}$	47.387 **
$\alpha_{S}^{+}$	0.046 **
$\alpha_{S}^{-}$	0.016 **
$\gamma_{S}$	2.282
τ	2.676

** strong evidence; *** extreme evidence.

## Appendix F

**Table A7:** Pearson correlation coefficients between model parameters and conditional error probabilities as well as participants’ demographic, clinical, and psychological characteristics.

	Healthy Control Participants							Parkinson’s Disease Patients						
	$\alpha_{C}^{+}$	$\alpha_{C}^{-}$	$\gamma_{C}$	$\alpha_{S}^{+}$	$\alpha_{S}^{-}$	$\gamma_{S}$	τ	$\alpha_{C}^{+}$	$\alpha_{C}^{-}$	$\gamma_{C}$	$\alpha_{S}^{+}$	$\alpha_{S}^{-}$	$\gamma_{S}$	τ
Perseveration errors	−0.55 **	−0.92 ***	−0.66 ***	−0.32	0.34	0.08	0.52 **	−0.63 *	−0.91 ***	−0.44	0.34	0.54	0.30	0.63 *
Set-loss Errors	−0.94 ***	−0.70 ***	−0.61 ***	−0.21	0.07	−0.04	0.80 ***	−0.96 ***	−0.69 **	−0.57 *	0.67 *	0.22	0.30	0.88 ***
Age (years)	−0.40 *	−0.30	−0.35	−0.06	−0.06	0.15	0.42 *	−0.50	−0.42	−0.46	0.39	−0.02	0.32	0.42
Education (years)	0.16	0.07	−0.03	−0.05	0.10	−0.21	−0.05	0.62 *	0.34	0.40	−0.54	−0.18	−0.05	−0.55
Disease duration (years)								−0.47	−0.10	−0.05	0.51	−0.03	0.09	0.33
UPDRS III ‘on’								−0.18	0.20	0.02	0.14	−0.41	−0.20	0.24
UPDRS III ‘off’								−0.20	0.47	0.32	0.14	−0.14	−0.06	0.19
LEDD								−0.29	−0.01	0.06	0.23	−0.23	−0.24	0.19
MoCA (cognitive status)	0.42 *	0.34	0.23	−0.21	0.17	0.12	−0.39	0.03	0.45	0.16	0.00	−0.05	−0.11	−0.16
WST (premorbid intelligence)	0.33	0.28	0.21	0.07	0.01	−0.08	−0.40	0.36	0.31	0.15	0.02	0.15	0.09	−0.43
AES (apathy)	−0.14	−0.14	−0.03	−0.21	0.04	0.10	0.02	−0.50	0.08	0.05	0.10	−0.32	−0.02	0.48
BDI-II (depression)	−0.14	−0.21	−0.21	−0.04	0.18	−0.05	0.09	−0.02	0.41	0.19	−0.37	−0.38	−0.17	0.04
BSI-18 (psychiatric status)	−0.23	−0.22	−0.26	−0.13	0.07	−0.02	0.16	−0.38	−0.11	−0.25	0.20	0.15	0.52	0.27
Anxiety	−0.24	−0.17	−0.23	−0.15	0.11	0.11	0.18	−0.02	0.05	−0.14	−0.14	0.08	0.35	−0.07
Depression	−0.18	−0.22	−0.23	−0.09	0.05	−0.20	0.08	−0.50	−0.20	−0.32	0.03	0.03	0.23	0.40
Somatization	−0.21	−0.20	−0.25	−0.11	0.06	0.07	0.17	−0.39	−0.11	−0.20	0.39	0.19	0.60 *	0.29
SF-36 (health status)	0.23	0.31	0.26	0.06	−0.04	−0.03	−0.26	0.28	−0.11	0.22	−0.13	0.24	−0.41	−0.19
Physical functioning	0.21	0.20	0.16	0.19	0.02	0.01	−0.22	0.14	−0.32	−0.03	−0.31	0.00	−0.48	−0.06
Physical role functioning	0.23	0.41 *	0.29	0.11	−0.07	−0.03	−0.29	−0.02	−0.40	−0.02	−0.11	0.45	−0.28	0.07
Bodily pain	0.25	0.29	0.23	0.26	−0.17	−0.20	−0.23	0.27	0.15	0.47	−0.29	−0.12	−0.44	−0.13
General health perception	0.24	0.30	0.27	0.16	−0.10	−0.21	−0.31	0.23	−0.05	0.15	−0.23	0.18	−0.58	−0.20
Vitality	0.03	0.00	0.03	−0.06	−0.04	0.01	−0.06	0.18	−0.24	−0.03	−0.02	0.30	−0.30	−0.18
Social role functioning	0.29	0.31	0.18	0.04	−0.05	0.00	−0.24	0.22	−0.18	−0.22	0.27	0.47	0.25	−0.25
Emotional role functioning	0.10	0.22	0.21	−0.14	0.05	0.07	−0.10	0.35	0.25	0.34	0.19	0.01	0.14	−0.32
Mental health	0.14	0.17	0.21	−0.15	0.07	0.05	−0.16	0.19	0.12	0.25	0.17	0.27	−0.13	−0.12
BIS-brief (impulsiveness)	−0.17	−0.17	−0.07	−0.17	0.06	0.22	0.08	−0.12	0.45	−0.02	−0.05	−0.24	−0.03	−0.01
DII (impulsivity)														
Functional	0.03	0.06	0.09	0.04	−0.07	0.06	0.13	−0.06	−0.31	−0.26	0.18	0.06	−0.21	0.04
Dysfunctional	−0.23	−0.11	−0.01	0.06	−0.19	−0.04	0.30	−0.19	0.28	−0.03	−0.22	−0.27	−0.32	0.10
QUIP-RS (impulse control)	−0.03	0.23	0.27	0.02	−0.14	−0.23	−0.04	0.13	0.45	0.37	−0.11	−0.08	0.07	−0.17
SPQ (schizotypal traits)	−0.12	−0.27	−0.12	−0.33	0.30	−0.04	0.03	−0.28	0.04	0.00	−0.26	0.01	−0.21	0.26
Interpersonal	−0.14	−0.33	−0.15	−0.25	0.16	0.00	0.09	−0.31	0.07	0.10	−0.11	0.11	0.16	0.29
Cognitive-perceptual	0.10	0.17	0.12	−0.15	0.49 **	0.00	−0.24	0.05	0.11	0.09	−0.33	0.10	−0.49	−0.05
Disorganized	−0.19	−0.35	−0.20	−0.38	0.11	−0.13	0.13	−0.34	−0.10	−0.21	−0.19	−0.19	−0.27	0.33

Individual-level model parameters were computed as modes of posterior distributions obtained from Equation (A13). Posterior distributions of individual-level model parameters and conditional error probabilities were pooled across session (and medication) effects. Disease duration was defined as the difference between the year of testing and the year of Parkinson’s disease symptom onset in the medical records; UPDRS III = Unified Parkinson’s Disease Rating Scale-part III; LEDD = Levodopa Equivalent Daily Dosage (mg per day) [102]; MoCA = Montreal Cognitive Assessment [67]; WST = Wortschatztest [103]; AES = Apathy Evaluation Scale [104]; BDI-II = Beck Depression Inventory-II [105]; BSI- 18 = Brief Symptom Inventory (18-item version) [106]; SF-36 = Short Form Health Survey [107]; BIS-Brief = Barratt Impulsiveness Scale (8-item version) [108]; DII = Dickman Impulsivity Inventory [109]; QUIP-RS = Questionnaire for Impulsive-Compulsive Disorders in Parkinson’s Disease—Rating Scale [110]; SPQ = Schizotypal Personality Questionnaire [111]; * substantial evidence; ** strong evidence; *** extreme evidence.
